# The Receptor for Advanced Glycation Endproducts (RAGE) Contributes to Severe Inflammatory Liver Injury in Mice

**DOI:** 10.3389/fimmu.2020.01157

**Published:** 2020-06-03

**Authors:** Toni Weinhage, Timo Wirth, Paula Schütz, Philipp Becker, Aloys Lueken, Boris V. Skryabin, Helmut Wittkowski, Dirk Foell

**Affiliations:** ^1^Department of Pediatric Rheumatology and Immunology, University of Münster, Münster, Germany; ^2^Department of Neurology With Institute of Translational Neurology, University Hospital Münster, Münster, Germany; ^3^Core Facility of Transgenic Animal and Genetic Engineering Models (TRAM), University of Münster, Münster, Germany

**Keywords:** RAGE, sepsis, inflammatory liver injury, PAMPs, DAMPs

## Abstract

**Background:** The receptor for advanced glycation end products (RAGE) is a multiligand receptor involved in a number of processes and disorders. While it is known that RAGE-signaling can contribute to toxic liver damage and fibrosis, its role in acute inflammatory liver injury and septic multiorgan failure is yet undefined. We examined RAGE in lipopolysaccharide (LPS) induced acute liver injury of D-galN sensitized mice as a classical model for tumor necrosis factor alpha (TNF-α) dependent inflammatory organ damage.

**Methods:** Mice (*Rage–/–* and C57BL/6) were intraperitoneally injected with D-galN (300 mg/kg) and LPS (10 μg/kg). Animals were monitored clinically, and cytokines, damage associated molecular pattern molecules (DAMPs) as well as liver enzymes were determined in serum. Liver histology, hepatic cytokines as well as RAGE mRNA expression were analyzed. Cellular activation and functionality were evaluated by flow cytometry both in bone marrow- and liver-derived cells.

**Results:** Genetic deficiency of RAGE significantly reduced the mortality of mice exposed to LPS/D-galN. Hepatocyte damage markers were reduced in *Rage–/–* mice, and liver histopathology was less severe. *Rage–/–* mice produced less pro-inflammatory cytokines and DAMPs in serum and liver. While immune cell functions appeared normal, TNF-α production by hepatocytes was reduced in *Rage–/–* mice.

**Conclusions:** We found that RAGE deletion attenuated the expression of pro-inflammatory cytokines and DAMPs in hepatocytes without affecting cellular immune functions in the LPS/D-galN model of murine liver injury. Our data highlight the importance of tissue-specific RAGE-signaling also in acute inflammatory liver stress contributing to sepsis and multiorgan failure.

## Introduction

Management of severe sepsis is still one of the major challenges in medicine today. It is associated with high mortality rates of over 50%. In particular, preventive strategies and effective treatment options for life-threatening multiorgan failure and acute septic shock syndromes remain relatively scarce (1–3).

Pathogen associated molecular patterns (PAMPs) such as lipopolysaccharides (LPS) are critically involved in the pathogenesis of bacterial sepsis. During the early response to infection, dysregulation and overproduction of acute-phase cytokines correlate with morbidity and lethality in humans as well as in animal models of sepsis. Rodents are known to be more than 1,000-fold less sensitive to LPS compared to humans and thus, they are often sensitized by pretreatment with the amino sugar D-galactosamine (D-galN) (4). Injection of D-galN together with LPS is reported to result in a sensitization of up to 10,000-fold toward the initial stimulus. D-galN is metabolized exclusively in the liver and causes a selective depletion of uridine nucleotides culminating in hepatic transcriptional block (5). However, although D-galN can severely sensitize mice to the lethal effects of LPS, it is hardly toxic on its own (4). Moreover, it has been shown that LPS challenge in D-galN-sensitized mice primarily causes fulminant hepatitis and only accounts to a secondary multiple organ dysfunction syndrome (MODS) (6).

In the LPS/D-galN model of hepatic injury, the pro-inflammatory cytokine tumor necrosis factor α (TNF-α) seems to be both necessary and sufficient to mediate lethality due to the consecutive septic shock (5, 7, 8). However, the exact mechanisms of hepatic damage and the cause of lethality have not been resolved at the molecular level. Although neutralization of TNF-α or interleukin 1 β (IL-1β) did not reduce mortality in sepsis trials (9, 10), experimental and clinical data have shown that these proinflammatory cytokines with specific relevance for innate immune functions are important mediators of severe sepsis (11, 12).

However, sepsis is not limited to the interaction of immune cells with pathogens but is also driven by endogenous mediators collectively termed “damage associated molecular patterns” (DAMPs), danger signals, or “alarmins.” Sepsis-like disease can even develop in sterile conditions leading to a process collectively also termed “systemic inflammatory response syndrome” (SIRS). The receptor for advanced glycation end products (RAGE) is a multiligand pattern recognition receptor (PRR) recognizing a wide range of DAMPs upon inflammation and injury (13). Ligands include advanced glycation endproducts (AGEs), high mobility group box 1 (HMGB1), amyloid-β peptide, β2-integrins and S100/calgranulins, especially the S100A8/S100A9 heterocomplex (14). It is well-established that engagement of RAGE leads to release of pro-inflammatory cytokines via nuclear factor-kappa B (NF-κB) mediated pathways (15). Except for the lung, RAGE is expressed at relatively low basal levels in healthy tissues (16, 17). Under pathophysiological conditions like in diabetes, inflammation or neurodegeneration, RAGE can be dramatically upregulated (14, 18). This receptor is also expressed on hepatic stellate cells, epithelial hepatocytes and Kupffer cells (19, 20). However, its contribution to inflammatory liver disease is still an unresolved issue (21).

RAGE mediates function and migration of monocytes and neutrophils in a DAMP-dependent manner (22–24). RAGE is also known to protect against bacterial pneumonia (25). In addition, a role in the progression of gut and joint inflammation has been shown (26, 27). It thus appears likely that this receptor plays a role in DAMP-mediated hyperinflammation following tissue injury. Initial results have shown that RAGE is particularly involved in innate immune responses during sepsis not only at the initiating steps but also in the phase of perpetuation (28). While initially adaptive immune functions have been described unaffected, later a role of RAGE also in inflammatory activation of antigen-primed T cells has been proposed (29). However, the role of RAGE during infection and sepsis remains complex, as it may contribute to the protection against bacterial dissemination on the one hand and to the progression of inflammatory organ damage on the other hand (30–32).

To better understand the RAGE axis during hepatic inflammation and sepsis, we used the murine LPS/D-galN induced liver injury model in RAGE gene deficient (*Rage–/–*) and C57BL/6 wildtype (wt) mice (4). We found that deletion of RAGE attenuated the hepatic expression of pro-inflammatory cytokines, while the activation of immune cells was unaffected. More importantly, *Rage–/–* mice also displayed improved survival after challenge with LPS/D-galN doses lethal for wt littermates. Our findings in this highly liver-specific inflammatory injury model highlight the importance of RAGE in pathologic conditions where both DAMP- and PAMP-related signaling triggers potentially fatal MODS.

## Materials and Methods

### Animals

*Rage–/–* mice were generated as described in detail in the Supplementary Methods. All animals were housed together with C57BL6/J wildtype mice in the animal facility of the University Hospital Münster under standard pathogen-free conditions. All animal procedures were conducted in accordance with the German Animal Welfare Act and approved by the responsible State Agency (LANUV NRW reference No. 84-02.04.2014.A223).

### D-galN/LPS Induced Inflammatory Liver Injury

Animals were co-injected with 300 mg/kg D-galN and 10 μg/kg LPS from *E. coli* 055:B5 (both from Sigma-Aldrich, Taufkirchen, Germany), diluted in sterile pyrogen-free saline (B. Braun Melsungen AG, Melsungen, Germany). Animals were monitored for survival until times indicated or until appropriate endpoints were reached and then sacrificed by CO_2_ inhalation and cervical dislocation.

### Preparation of Murine Liver Specimens

Immediately after death, the abdomen was opened and blood was collected from the heart. Infusion of phosphate buffered saline (PBS) containing EDTA and HEPES was initiated into the right ventricle to blanch the liver. After bleaching, the liver was perfused with Dulbecco's modified eagle medium (DMEM, low glucose) containing penicillin/streptomycin, HEPES and type 4 collagenase. The portal vein was cut and also perfused with digestion medium. After removal of the gallbladder, the liver was carefully excised from the abdomen. The organ was minced into small pieces with sterile surgical scissors. The minced pieces were further digested in a thermostatic bath at 37°C, stirring (100 rpm) for 10 min. The preparation was homogenized and forced through a 70 μm cell strainer. The cells were resuspended, centrifuged gently at 50 x g without brake for 2 min at room temperature. The cells were transferred, followed by further washing steps and lysis of remaining erythrocytes. Finally, the complete liver cells were counted and resuspended in supplemented DMEM containing FCS. Hepatocytes were cultured in type 1 collagen-coated plates, while immune cells were further processed as described below. In further experiments, the liver was directly excised, transferred to formalin-alcohol fixatives and embedded into paraffin for later immunohistochemistry (see below).

### Isolation of Intrahepatic Immune Cells (IHICs)

Single cell suspensions of IHICs were prepared by mechanical dissociation as outlined above and further processed as previously described (33). For intracellular cytokine staining experiments, cells were further enriched using MojoSort Mouse CD45 Nanobeads (BioLegend, San Diego, CA, USA). IHICs were plated to isolate Kupffer cells by adhesion at 37°C for 30 min. The cells were stimulated as indicated and subsequently analyzed by flow cytometry.

### Processing of Blood and Isolation of Bone-Marrow-Derived Cells

Blood was collected from retro-orbital sinus vein into heparinized 1.5 ml microcentrifuge tubes for the measurement of disease markers in monitoring experiments lasting up to 4 h. Neutrophils were isolated using the EasySep™ Mouse Neutrophil Enrichment Kit (Stemcell Technologies, Cologne, Germany). Bone marrow-derived cells were handled as described previously (34). Cells were centrifuged (350 × g, 5 min, RT) and remaining red blood cells were lysed. For neutrophil isolation, the cell suspension was transferred into sterile 5 ml FACS tubes and neutrophils were isolated using the EasySep Mouse Neutrophil Enrichment Kit according to the manufacturer's protocol. For bone marrow derived macrophage (BMDM) isolation, cells were initially incubated at 37°C and 5% CO_2_ to allow for attachment. Attached cells were cultured in 20% L929 cell supernatant conditioned DMEM containing 2 mM L-glutamine, 0.1 mM non-essential amino acids (all Invitrogen, Karlsruhe, Germany), penicillin (100 U/mL), streptomycin (100 mg/mL), and 10% FCS (all Biochrom). Cells were plated on petri-culture dishes in DMEM containing 10% FCS. After 30 min incubation at 37°C, non-adhering cells were washed away. The remaining cells were detached using ice-cold 5 mM EDTA PBS buffer. BMDMs were fully differentiated and ready for use after 7 days of culture.

### Analysis of Serum and Culture Supernatants

Determination of cytokine concentrations in culture supernatants and serum was performed using a multiplex assay (LEGENDplex™ Mouse Inflammation Panel; BioLegend). ALT and AST levels were measured using Amplite™ fluorimetric aspartate aminotransferase (AST) and alanine aminotransferase (ALT) assays (AAT Bioquest, Sunnyvale, CA, USA). S100A9 concentrations in serum were determined using the mouse S100A9 DuoSet ELISA (R&D Systems, Wiesbaden, Germany). HMGB1 levels were assessed by a HMGB1 ELISA Detection Kit (Chondrex Inc., Redmond, WA, USA).

### Flow Cytometry

Cells were stained with 1 μg/ml fluorochrome-conjugated antibodies as described previously (34). The used monoclonal antibodies are given in Supplementary Table 1. Intracellular staining of cytokines was performed using fixation/permeabilization buffer (eBioscience, San Diego, CA, USA). Prior to intracellular staining with anti-TNF antibody, cells were stimulated with 1X cell stimulation cocktail (plus protein transport inhibitors) (eBioscience) for 3 h and incubated with rat anti-mouse CD16/CD32 antibodies to block non-specific binding of immunoglobulin to Fc receptors (eBioscience). Samples were analyzed on FACSCanto (BD Immunocytometry Systems, Heidelberg, Germany) or CyFlow® Space (Sysmex Partec GmbH, Görlitz, Germany) flow cytometers.

### Functional Characterization of Phagocytes

Phagocytic capacity and reactive oxygen species (ROS) generation of monocytes and neutrophils were analyzed as described previously (34). Briefly, phagocytosis of BMDM was determined by incubation with fluorescein labeled *E. coli* particles for 1 h. Neutrophils were incubated with pHrodo® Green *E. coli* BioParticles® (Thermo Fisher Scientific, Waltham, MA, USA) for 1 h at 37°C. To induce ROS production, BMDM or neutrophils were stimulated with LPS (10 ng/mL; Sigma-Aldrich) for 4 or 1 h at 37°C in the presence of 15 μM dihydrorhodamine 123 (Merck, Darmstadt, Germany) for the final 15 min, respectively. For flow cytometry-based direct quantification of neutrophil extracellular traps (NETs) release from neutrophils, 1 × 10^5^ freshly isolated murine bone marrow derived neutrophils in RPMI 1640 (without Phenol Red) were transferred into a sterile 5 ml FACS tube and stimulated with 4 μM ionomycin. After fixation and blocking of unspecific binding sites, the cells were stained for citrullinated histone H3 (citH3) and myeloperoxidase (MPO) to identify NETs. As secondary antibody for citH3, polyclonal AF647-conjugated donkey anti-rabbit IgG antibody was used (Biolegend). Samples were analyzed on a CytoFLEX flow cytometer (Beckman Coulter, Krefeld, Germany).

### Real-Time Quantitative PCR

Gene expression analysis was performed as previously described (35). Real-time quantitative PCR was performed on a CFX384 Touch real-time PCR detection system (Bio-Rad Laboratories, Munich, Germany). Relative mRNA levels were determined by normalization to the housekeeping gene(s) RPS9, HPRT, and GAPDH. Primers are listed in Supplementary Table 2.

### Immunohistochemistry and Immunofluorescence Microscopy

Livers were excised and transferred to formalin-alcohol fixatives immediately. Paraffin embedded tissues were cut at 3–5 μm, de-paraffinized, treated with proteinase K and blocked with 10% BSA in PBS containing 0.05% Tween and then incubated overnight at 4°C with primary antibodies diluted in PBS containing 2% BSA. S100A8, S100A9 (rabbit-anti-S100A8 or -S100A9, Thermo Fisher Scientific) and HMGB-1 (rabbit-anti-HMGB1, Abcam) were stained to localize DAMPs in liver sections, using goat-anti-rabbit biotinylated secondary antibodies and peroxidase. Slides were counterstained with haematoxylin eosin (HE) and analyzed using a cumulative damage scoring system (Supplementary Table 3) by an observer blind to the treatment groups. For immunofluorescence analyses, sections were stained with primary antibodies followed by incubation with fluorochrome-conjugated secondary antibodies. DAPI was used for staining of nuclei. All stainings were visualized using an AxioObserver.Z1 microscope (Carl Zeiss, Oberkochen, Germany).

### Statistical Analysis

Data are expressed as mean ± SEM unless stated otherwise. For non-normally distributed variables Mann-Whitney tests were used. Otherwise data was analyzed by one-way ANOVA or repeated measures ANOVA with Fisher's LSD test, as appropriate. Survival was analyzed using Log-rank (Mantel-Cox) tests. Statistical differences were considered significant when *p* was < 0.05.

## Results

### RAGE Deficiency Attenuates LPS/D-galN Induced Mortality

**Figure 1:**
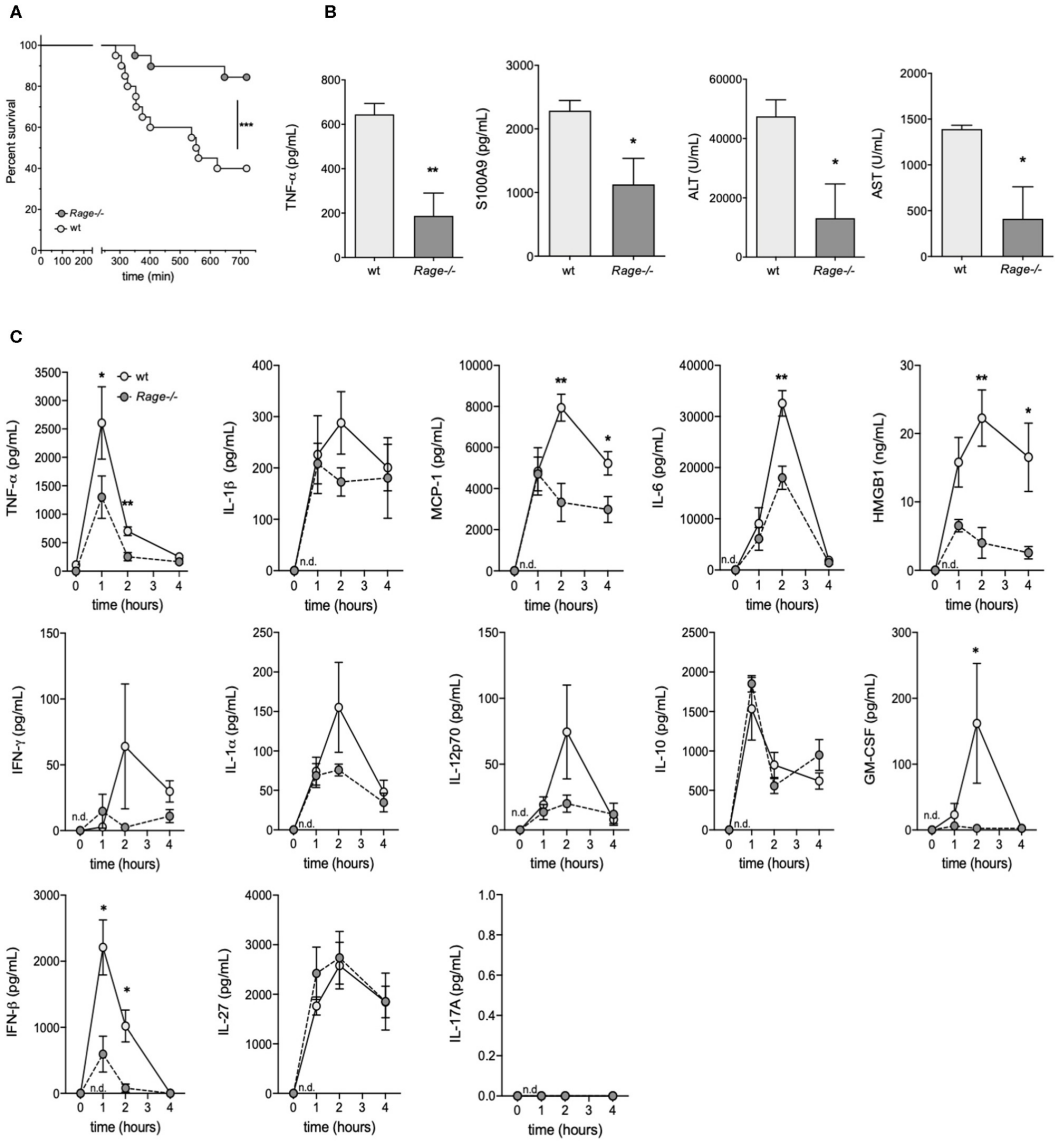
Outcome of *Rage–/–* and wildtype (wt) mice after LPS/D-galN challenge. Mice were injected i.p. with 300 mg/kg D-galN and 10 μg/kg LPS. **(A)** Mouse survival post-injection is shown (*n* = 20, respectively, for each genotype in 3 independent experiments). **(B)** Serum levels of proinflammatory cytokines TNF-α, S100A9 as well as liver enzymes ALT and AST were measured immediately after mice succumbed to LPS/D-galN induced inflammatory liver injury (*n* = 3–4). **(C)** Wildtype (wt, light gray circles) and *Rage–/–* mice (dark gray circles, *n* = 4 each time point) were sacrificed after LPS/D-galN challenge at the time points indicated. Serum levels of TNF-α, IL-1β, MCP1, Interleukin-6, HMGB-1, IFN-γ, IL-1α, IL-12p70, IL-10, GM-CSF, IFN-β, IL-27, and IL-17A. The latter was not detectable. Data are expressed as the mean (± SEM). Statistical analysis was performed using Log rank (Mantel-Cox) **(A)** or Mann-Whitney test **(B,C)** comparing wt to *Rage–/–* mice. ^*^p < 0.05, ^**^p < 0.01, ^***^p < 0.001.

*Rage–/–* and C57 wildtype mice were intraperitoneally (i.p.) injected with D-galN (300 mg/kg) and LPS (10 μg/kg). All animals showed clinical signs of disease such as decreased locomotion and reduced grooming as early as 4–6 h after challenge. However, there was a significant reduction of mortality in *Rage–/–* mice. The experiments had to be terminated after 12 h. Whereas, only 7/20 (35%) of the wildtype animals were still alive at this time point, 16/20 (80%) of *Rage–/–* mice still survived up to 720 min (Figure 1A).

### Disease Markers Are Decreased in Rage–/– Mice at the End of the Experiment

As a means to examine the overall disease burden in the challenged mice, the concentrations of inflammatory biomarkers and liver enzymes were determined in blood obtained from LPS/ D-galN-challenged surviving mice which had to be sacrificed after 12 h (at the end of the experimental observation period). All analyzed biomarkers of disease were significantly lower in *Rage–/–* mice compared to wildtype mice (Figure 1B). To measure hepatocyte damage, we examined serum ALT and AST activity. ALT/AST levels were massively increased compared to untreated control animals in wildtype but not in RAGE-/- mice. Administration of LPS or D-galN alone produced a moderate or no increase in serum ALT and AST levels (data not shown). As the LPS/D-galN liver injury model is known to depend on TNF-α, levels of this cytokine were analyzed. Furthermore, S100A9 is an important DAMP molecule and a RAGE ligand that is also known to promote lethal endotoxin-induced shock (36). Consistently, S100A9 serum levels were increased after LPS/D-galN injection and, more importantly, significantly reduced in *Rage–/–* mice compared to wildtype mice.

### Monitoring of Inflammatory Cytokines/Chemokines Early After Challenge

As not all animals survived up to the final time point and it seemed conceivable that markers even peak before the end of the observation period, we consecutively monitored the levels of inflammatory cytokines/chemokines in serum of *Rage–/–* and wildtype mice to account for the sequence of events preceding massive liver injury and death (Figure 1C). At early time points after challenge, TNF-α levels rose very rapidly in the circulation, reaching a peak at 1 h. Bursts of IL-6, IL-1β, HMGB-1 and the chemokine MCP-1 at 2 h followed the very early rise in TNF-α. Other cytokines also showed a rise after challenge. The response seen with most serum factors was attenuated in *Rage–/–* mice (Figure 1C). Administration of LPS alone induced similar responses of cytokines and chemokines, but D-galN alone did not generate a significant increase *in vivo* (data not shown), confirming LPS to be required as an inflammatory cytokine/chemokine inducer. However, more importantly, the rise of pro-inflammatory molecules was significantly reduced in *Rage–/–*animals, affirming the impact of RAGE signaling on their *in vivo* production.

### Reduced LPS/D-galN-Induced Hepatic Injury in Rage–/– Animals

**Figure 2:**
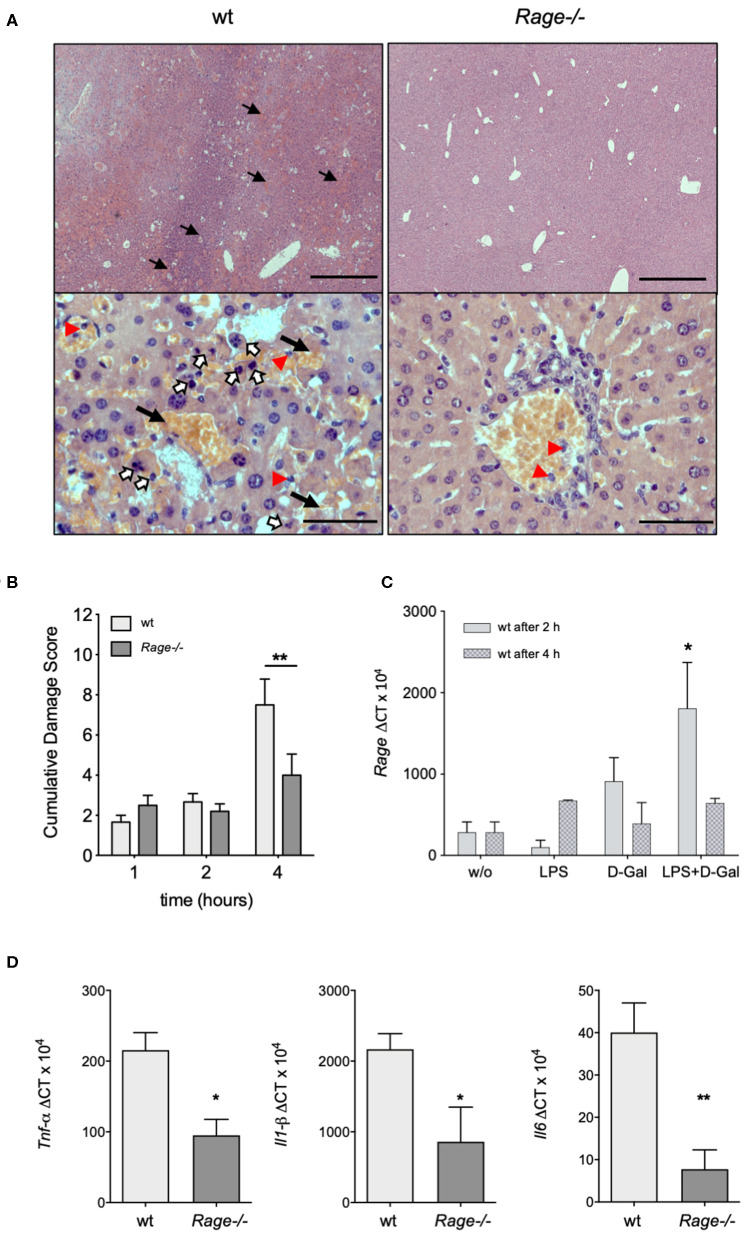
Hepatic pathology after LPS/D-galN challenge. **(A)** Histopathology in LPS-induced liver damage of D-galN sensitized *Rage–/–* and wildtype (wt) mice. H&E staining of wt and *Rage–/–* livers at 50x (*upper panel, scale bar* = *500* μ*m*) and 400x (*lower panel, scale bar* = *50* μ*m*) original magnifications. Massive necrosis, associated with intralobular hemorrhage (arrows), destruction of hepatic architecture as well as hepatocellular apoptosis (open arrows) and infiltration of neutrophils (red arrowheads) was visible in WT animals. Slight hepatic necrosis with minor inflammatory cell infiltration was observed in *Rage–/–* mice. **(B)** Quantification of LPS/D-galN induced inflammatory liver injury using a damage score that combines grading of hepatocellular necrosis, small vacuolisation and/or cell lysis, accumulation of erythrocytes in the sinusoids and neutrophilic infiltration at the time points indicated (*n* = 4 each time point). **(C)** *Rage* mRNA expression in wt liver tissue at 2 and 4 h after LPS/D-galN injection (*n* = 3). **(D)** Liver mRNA expression of pro-inflammatory cytokines *Tnf*-α, *Interleukin-1 beta*, and *Interleukin-6* in mice after 4 h of LPS/D-galN induced inflammatory liver injury (*n* = 4–5). Data represent the mean + SEM of the results obtained from two independent experiments. Statistical analysis was performed using Mann-Whitney test comparing wildtype to *Rage–/–* mice **(B,D)** or untreated to challenged wildtype mice **(C)**, ^*^p < 0.05, ^**^p < 0.01. LPS, lipopolysaccharide; D-Gal, D-galN (D-galactosamine).

Next, we aimed to analyse whether the more prominent clinical and laboratory-proven deterioration of wildtype animals after LPS/D-galN administration was actually due to liver injury. For this purpose, the liver was explanted from mice that had been challenged with LPS/D-galN for up to 4 h. Already macroscopically, the challenged mice showed hepatic enlargement and hemorrhage, which appeared more severe in wildtype animals. Microscopic evaluation of the hepatic histology revealed characteristic changes of organ architecture and confirmed massive apoptosis and hemorrhagic liver necrosis of all wildtype mice. Affected animals exhibited a massive neutrophil infiltration (Figure 2A). In contrast, *Rage–/–* mice showed a minimal infiltration of neutrophils. There was only little injury and minor hemorrhagic necrosis in livers from *Rage–/–* mice, resulting in a significantly reduced cumulative damage score (Figure 2B).

**Figure 3:**
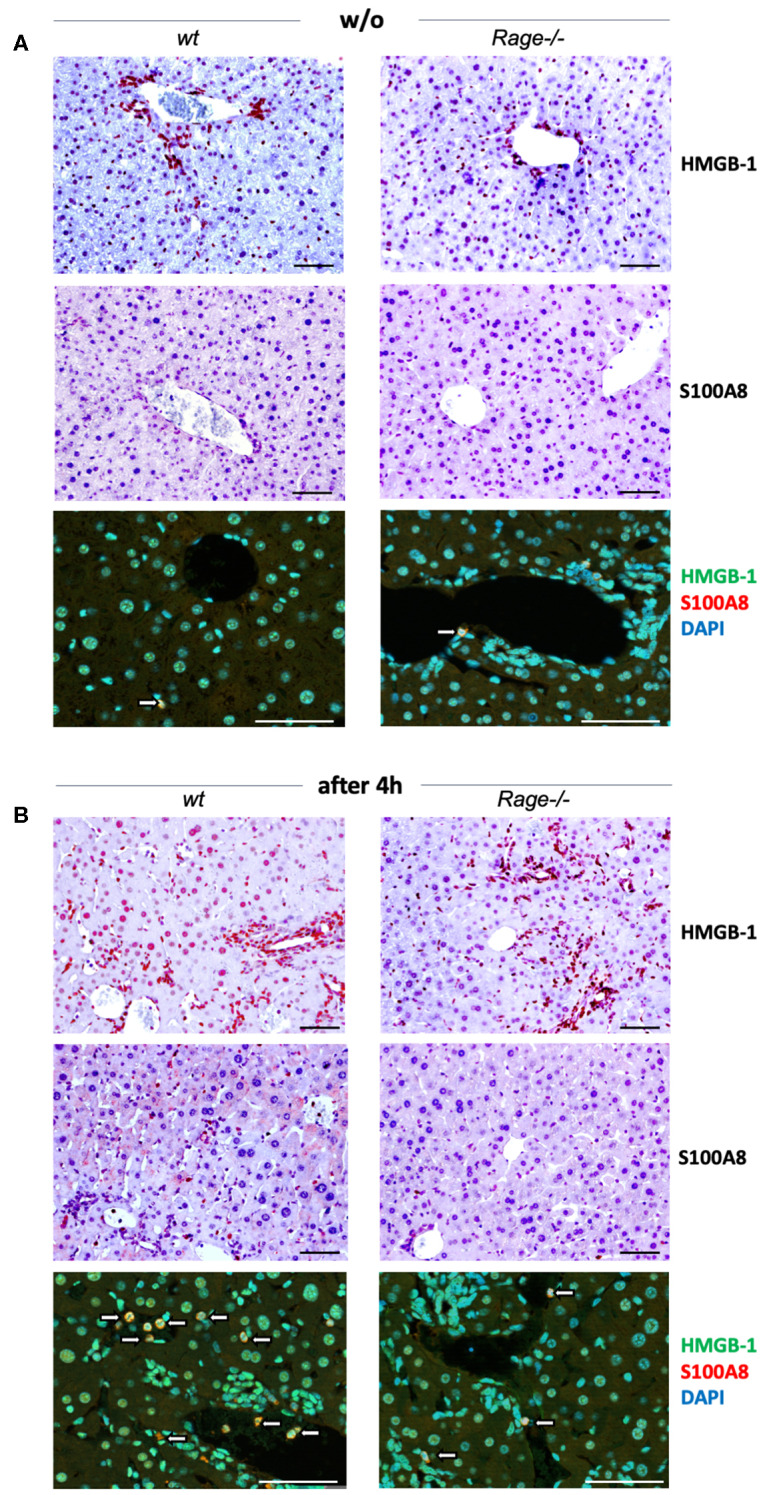
Localization of DAMPs in hepatic tissue. **(A)** Analyses in non-challenged wildtype (wt) or *Rage–/–* mice. Immunohistochemical staining at 200x (*upper panels, scale bars 50* μ*M*) confirmed the expression of HMGB-1 in parenchymal and non-parenchymal hepatic cells, which was restricted to the nucleus. S100A8 was only found in the cytoplasm of few Kupffer cells (white open arrows). Immunofluorescence analyses at 400x (*lower panel, scale bars 50* μ*M*) confirmed the distinct staining patterns of HMGB-1 (FITC, green) and S100A8 (Alexa fluor 488, red). DAPI (blue) was used to counterstain nuclei. **(B)** In the liver of mice 4 h after LPS/D-galN injection, there was a stronger staining of HMGB-1, but without significant translocation from the nucleus into the cytoplasm within the short timeframe. In addition, more infiltrating S100A8-positive myeloid cells were present (white open arrows). When compared to *Rage–/–* animals, the expression of DAMPs was more pronounced in damaged liver sections from wt mice.

Activation of RAGE can perpetuate inflammation by NF-κB dependent sustained RAGE expression, which creates a positive-feedback loop amplifying its own expression (14, 37). To account for this possibility also in inflammatory liver injury, we measured mRNA expression of RAGE in liver extracts 2 and 4 h after injection of LPS and/or D-galN (Figure 2C). Interestingly, sole D-galN injection induced a rise in RAGE transcripts that was further amplified by simultaneous LPS treatment rapidly after challenge, which may further propagate liver injury in this model. We were not able to analyse RAGE protein expression in different cells, such as hepatocytes and Kupffer cells. However, we found that in specimens from the liver after 4 h, organ cytokine mRNA (TNF-α, IL-1β, IL-6) production was significantly lower in *Rage–/–* liver specimens compared to wildtype mice (Figure 2D). In addition, we found that the expression of DAMPs S100A8 and HMGB-1 correlated with the cell infiltration and tissue damage after challenge (Figure 3), which was less extensive in *Rage–/–* mice. HMGB-1 was expressed in all parenchymal and non-parenchymal cells with a predominant nuclear staining pattern, while S100A8 was restricted to the cytoplasm of infiltrating immune cells and not present in hepatocytes.

### Rage–/– Bone Marrow-Derived Monocytes and Neutrophils Are Not Functionally Impaired

**Figure 4:**
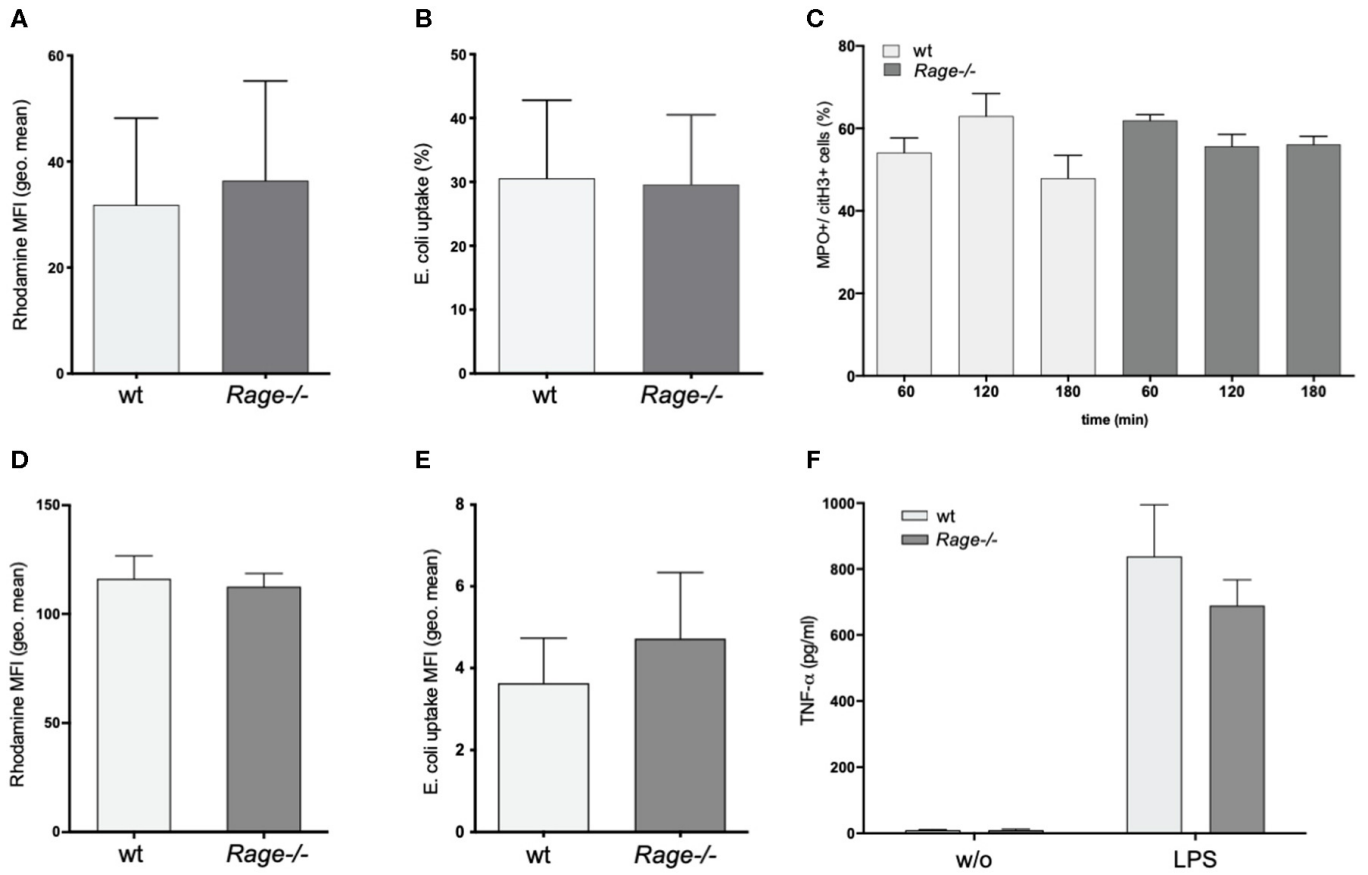
Functional characterization of neutrophils and bone marrow derived monocytes/macrophages (BMDM). **(A)** ROS production of neutrophils from wildtype (wt) or *Rage–/–* mice after 1 h. **(B)** Phagocytosis of FITC-labeled *E. coli* by neutrophils after 1 h. **(C)** NETosis of neutrophils after treatment with ionomycin (4 μM) for 60–180 min. **(D)** ROS production of BMDM after 1 h. **(E)** Phagocytosis of FITC-labeled *E. coli* by BMDM after 1 h. **(F)** Release TNF-α by BMDM into the cell culture supernatant after 6 h without (w/o) or with LPS (100 ng) stimulation. Data are expressed as the mean + SEM. MFI, mean fluorescence intensity; ROS, reactive oxygen species.

A striking feature of the aggravated hepatic disease in wildtype mice was the prominent neutrophil infiltration. Since neutrophils are major producers of ROS and involved in destructive tissue damage when over-activated, we evaluated the functional properties of isolated CD11b+Ly6G+ granulocytes from blood and bone marrow of *Rage–/–* and wildtype mice. ROS production or phagocytosis by granulocytes did not seem to depend on the presence of RAGE (Figures 4A,B). In addition, NET formation is a mode of neutrophils to react to stimuli, expose pro-inflammatory signals and counteract infectious invasion. In our experiments, there was a strong induction of NET formation with around 60% of netting cells after treatment with ionomycin. LPS stimulation had little effect on NETosis (data not shown). However, NETosis appeared equally effective in *Rage–/–* neutrophils (Figure 4C). RAGE has been implicated in the activation of mononuclear phagocytes. We found that adherence (not shown) and ROS production of BMDM from *Rage–/–* mice were unaltered (Figure 4D). Furthermore, the phagocytic activity toward Gram-negative bacteria was comparable (Figure 4E). Importantly, also *in vitro* TNF-α secretion of BMDM and mature peritoneal macrophages (not shown) were induced by incubation with LPS, but did not differ in *Rage–/–* cells, rendering it unlikely that peripheral monocyte and macrophage functionality is responsible for the observed phenotype in *Rage–/–* mice (Figure 4F).

### Liver Injury Is Associated With Cytokine Responses of Hepatocytes

**Figure 5:**
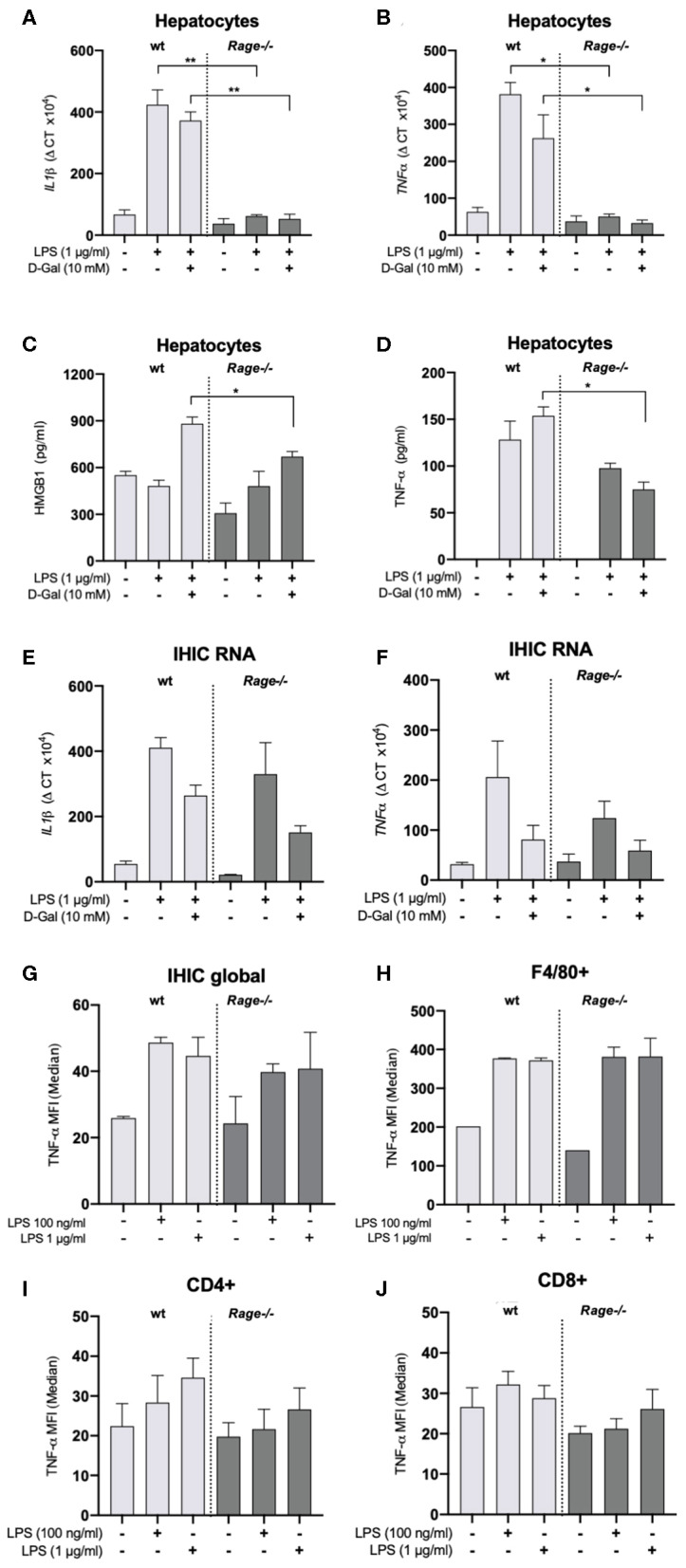
Characterization of hepatocytes and intrahepatic immune cells (IHICs). Primary liver cells were generated by established protocols. Cells were further isolated to separate hepatocytes and IHICs. **(A–D)** After culture for 4 days, hepatocytes were treated for 4 h with LPS (1 μg/ml) and D-galN (10 mM) or left untreated. Relative mRNA expression of *Il-1*β **(A)** and *Tnf*-α **(B)** in hepatocytes quantified by qRT-PCR. Gene expression levels were normalized to two housekeeper genes (*Hprt* and *Rps9*). **(C)** HMGB1 and **(D)** TNF-α concentrations released from hepatocytes into the supernatants after *in vitro* stimulation of hepatocytes with LPS and D-galN for 4 h. The stimulation with D-galN alone had no effect (not shown). Data are expressed as the mean ± SEM of two experiments (*n* = 4 per group). IHICs were isolated and subsequently stimulated with LPS for 4 h. Relative mRNA expression of *Il-1*β **(E)** and *Tnf*-α **(F)** in hepatocytes was quantified by qRT-PCR. Gene expression levels were normalized to two housekeeper genes (*Hprt* and *Rps9*). **(G,H)** IHICs were isolated and subsequently stimulated with LPS for 4 h in the presence of a protein transport inhibitor, followed by intracellular staining and FACS analyses to quantify intracellular TNF-α production. **(G)** Quantification of intracellular TNF-α production in IHIC isolated from *Rage–/–* and wildtype (wt) mice. **(H)** Quantification of intracellular TNF-α production in F4/80+/CD11b+ cells (resident macrophages/Kupffer cells). **(I,J)** Quantification of intracellular TNF-α production in CD4+ or CD8+ T lymphocytes. Data are depicted as mean ±SEM of two experiments (*n* = 4 per group). Data are expressed as the median ± SEM of two experiments (*n* = 4 per group). Statistical analysis was performed using one-way ANOVA; ^*^p ≤ 0.05, ^**^p ≤ 0.01. LPS, lipopolysaccharide; D-Gal, D-galN, D-galactosamine.

We have observed a strong production of cytokines, chemokines and DAMPs both systemically and locally in the liver, which was attenuated by RAGE-deficiency (Figures 1, 2). Monocytes/macrophages are supposed to be potent producers of cytokines such as TNF-α in response to innate immune activation as in sepsis. In this regard, it appeared rather surprising that innate immune functions were not different in the *Rage–/–* animals (Figure 4). In particular, TNF-α production was comparable between *Rage–/–* and wildtype animals. TNF-α is the crucial disease driver in the LPS/D-galN model (5). When cultured hepatocytes were stimulated with LPS/D-galN, an upregulation of cytokines and TNF-α as well as HMGB1 release was provoked, which was significantly lower in *Rage–/–* cells (Figures 5A–D). To further account for the exclusive liver environment, we isolated CD45+ IHICs (Figure 5E) and analyzed the intracellular TNF-α production after 4 h of stimulation with LPS. Neither intrahepatic phagocytes including Kupffer cells (Figure 5F) nor intrahepatic T lymphocytes (Figures 5G,H) revealed a strong reactivity independent of the genotype. In lymphocytes, a strong induction of TNF-α was seen when cells were stimulated with PMA/ionomycin, which was used as a positive control (Supplementary Figure 2). However, we conclude that a tissue- and cell specific mechanism involving RAGE signaling enhances TNF-α mediated damage and lethality.

## Discussion

Our data indicate that RAGE contributes directly to LPS/D-galN induced inflammatory liver damage by influencing hepatocyte activation and injury and not just as a secondary effect by regulating immune cell activation. RAGE serves as a receptor for non-enzymatically glycosylated molecules, leading to perpetuated inflammation and diabetic complications. It also binds endogenous danger signaling molecules mediating inflammation in non-infectious settings, thereby often priming cells for insults of pathogenic origin (38). RAGE is thus proposed to be involved in the pathogenesis of sepsis, mostly due to its ability of hyperactivation and perpetuation of inflammation. DAMPs such as S100 proteins, amyloid, or HMGB-1 are elevated in septic patients (28, 39, 40). These RAGE ligands have been shown to be important pro-inflammatory mediators promoting sepsis-related shock (36, 40–42). However, the release of RAGE ligands may target multiple cell types or receptors and thus the exact role of RAGE remains puzzling. The liver is an important site for immune surveillance and clearance of bacteria and their products. As such, liver cells express receptors for PAMPs and DAMPs, including members of the TLR system (43) but also RAGE.

Here, we demonstrate that genetic deletion of RAGE has a strong effect on morbidity and mortality and prevents full blown liver failure in a model of LPS-induced inflammatory liver injury after D-galN sensitization in mice. In line with our results, *Rage–/–* mice have been reported to be protected in a cecal ligation and puncture (CLP) polymicrobial sepsis model as well as in a model of systemic listeriosis (28, 44). Furthermore, in murine pneumococcal sepsis, it has been shown that RAGE deletion and administration of humanized anti-RAGE mAb could protect from lung inflammation (30, 45). LPS has also been proposed to bind directly to RAGE (46). In the same report, using an endotoxin dose of 50 mg/kg LPS, *Rage–/–* mice were reported to be spared significantly from LPS induced mortality, in concert with decreased TNF-α and HMGB-1 levels. Consistently, we also found a protective effect of genetic RAGE deletion in conjunction with diminished cytokine and DAMP production in *Rage–/–* animals. Importantly, we used a 1,000-fold lower LPS dose, rendering it unlikely that direct LPS-RAGE interaction explains the phenotype observed. Against this background, low-dose LPS on its own does not induce specific liver injury (6). With respect to HMGB-1 release and TNF-α production, *Rage–/–* mice have also been reported to respond less to lethal, however not liver-specific (high dose) endotoxin challenge (46). As we have demonstrated, the LPS/D-galN treatment results in a very acute and liver-specific inflammatory response that is characterized by a fast and massive increase of TNF-α, HMGB-1 and other pro-inflammatory cytokines and DAMP molecules.

Previous data showed that RAGE serves a complex role in bacterial sepsis. It can have a protective role by facilitating anti-infective immune reactions on the one hand, while on the other hand it can have a sepsis-promoting role by its pro-inflammatory function. As a consequence, increased bacterial dissemination was observed in *Rage–/–* mice in an *E. coli* peritonitis model accompanied by more hepatocellular injury and exaggerated systemic cytokine release. However, a much longer time course of 20 h was required until hepatic injury appeared (31). Other studies demonstrated that inhibition of RAGE during sepsis attenuates the systemic inflammatory response and organ damage. In line with these observations, our data provide further evidence of RAGE contributing to acute liver injury during inflammation.

Monocytes and macrophages are important mediators of inflammation but also contribute to tissue damage (47) and TNF-α production in the LPS/D-galN model (4, 5). In particular, in the LPS/D-galN model, Kupffer cells as resident liver macrophages may be involved in the pathogenicity (5). However, we found bone marrow derived monocytes/macrophages and primary myeloid cells isolated from the liver of *Rage–/–* mice functionally indistinguishable from wildtype controls. Besides the “cytokine storm” that we have observed as early as 1 h after LPS/D-galN challenge, we have also found elevated levels of the surrogate markers of hepatocellular damage ALT and AST, which were less prominent in *Rage–/–* mice compared to wildtype mice. In addition, the RAGE-ligands HMGB-1 and S100A9 were significantly lower expressed in *Rage–/–* mice. Interestingly, S100A9 has been reported to act as a necessary factor for the recruitment of neutrophils in acute and chronic liver injury (48). Undoubtedly, infiltration of neutrophils into the liver and their activation is crucial for host-defense and removal of cell debris. However, excessive neutrophil migration and over-activation can also cause tissue damage or even liver failure (49). In support of this concept, we histologically observed infiltration of neutrophils that was less pronounced in *Rage–/–* mice, consistent with the known HMGB-1/RAGE dependent, neutrophil-mediated injury amplification after acetaminophen induced liver damage (50). Engagement of neutrophils by surface Mac-1 receptors and through HMGB-1/RAGE interaction is known to stimulate NADPH dependent ROS production (51) that can also be a possible cause for cytotoxic necrosis when excessively generated (52). A role of murine RAGE for the phagocytosis of *Klebsiella pneumoniae* has been demonstrated (25). Importantly, we did not find intrinsic differences in ROS production and phagocytosis of *E. coli* between wildtype and *Rage–/–* neutrophils.

The model of D-galN sensitization is highly specific for the liver, as it targets biochemical carbohydrate metabolism pathways only present in hepatocytes (53). D-galN can deplete uridine phosphate pool from hepatic cells, resulting in a reduction of nucleic acid production and protein synthesis. However, in published studies, there was not a complete block of transcription when looking at rapidly inducible factors such as cytokines in liver tissue. Our results showed that LPS/D-galN significantly induced pro-inflammatory cytokines mRNA and protein expression in hepatocytes. We can extrapolate from published data, most of it using rat hepatocytes rather than murine cells, that RNA synthesis at D-galN doses as used by us is reduced starting after around 2 h, but not completely abolished (54–57). Our data have been generated in *ex vivo* cultures after stimulation lasting only 4 h. As is evident from our results, adding D-galN to LPS leads to some reduction of mRNA instead of a further increase (Figure 5). This does not exclude that *in vivo*, over time, D-galN blocks transcription by hepatocytes. To this end it remains to be shown whether liver metabolism of *Rage–/–* mice is comparable to wildtype animals. Nevertheless, we provide evidence that hepatocytes of *Rage–/–* mice specifically respond with reduced TNF-α production compared to control mice, suggesting a liver specific response that is dependent on RAGE signaling and that most likely also affects the pathology in the LPS/D-galN model. Hepatocytes are strong producers of a variety of soluble acute phase reactants and cytokines including TNF-α (58, 59). The cells express TLR4 and can react to LPS stimulation, but a RAGE-dependency is a new finding. Nevertheless, it has been demonstrated that parenchymal liver cell activation drives LPS-induced SIRS. Furthermore, LPS-induced murine systemic inflammation involves MCP-1 overexpression in activated parenchymal cells (60, 61). We also found early elevation of MCP-1 in mice after LPS/D-galN challenge, which was less pronounced on *Rage–/–* background.

There are some limitations to our studies. First, it is known that both epithelial hepatocytes and stellate cells express RAGE (19, 20). We were not able to further differentiate the exact cell types responsible for the significant reduction of inflammatory liver injury in *Rage–/–* mice. Our cultures of isolated liver cells could also still contain some remaining Kupffer cells, hence multiple hepatic cell components may have contributed to the overall liver-specific effects. Second, the exact mechanism whereby RAGE modulates signaling on the molecular level and the various targets of RAGE interactions remain unresolved. Third, we focus on TNF-α as the cytokine that is known to be responsible for lethality in the LPS/D-galN model. We cannot exclude that hepatocytes facilitate the disease processes by additional factors (e.g., chemokines) that can also support neutrophil infiltration as demonstrated in our experiments.

In summary, the present study demonstrates that genetic deletion of RAGE has a hepatoprotective effect against LPS/D-galN-induced TNF-α dependent acute liver damage in mice. We advance current knowledge regarding the mechanisms of how RAGE affects liver injury in the context of an acute inflammatory challenge. Although the etiology of MODS and sepsis is definitely multi-factorial, the liver may be amenable to therapeutic intervention by agents that target RAGE-dependent signaling pathways and interrupt the underlying disease mechanisms. In this way, our findings might prove to be useful in the development of future strategies that limit RAGE-related tissue damage in acute inflammatory liver injury and sepsis.

## Data Availability Statement

All datasets generated for this study are included in the article/Supplementary Material.

## Ethics Statement

The animal study was reviewed and approved by the state agency for nature, environment and consumer protection (Landesamt für Natur, Umwelt und Verbraucherschutz, LANUV NRW, Recklinghausen, Germany) under reference number 84-02.04.2014.A223.

## Author Contributions

TWe designed and supervised experiments, analyzed data, and wrote the manuscript. TWi supervised experiments, analyzed data, and wrote the manuscript. PS performed *in vitro* hepatocyte and neutrophil experiments. AL performed RAGE–/– cell characterization experiments. PB performed and analyzed initial survival experiments, immunoassays, and tissue real-time quantitative PCR. BS designed the RAGE target construct and performed and supervised experiments for the generation of *Rage–/–* mice. HW supervised experiments, interpreted data, and wrote the manuscript. DF designed the work, supervised experiments, analyzed and interpreted data, and wrote the manuscript. All authors were involved in writing the paper and approved the submitted manuscript.

## Conflict of Interest

The authors declare that the research was conducted in the absence of any commercial or financial relationships that could be construed as a potential conflict of interest.
