# Supplementary material for: The Receptor for Advanced Glycation Endproducts (RAGE) Contributes to Severe Inflammatory Liver Injury in Mice
Source: Front Immunol. 2020 Jun 3;11:1157. doi: 10.3389/fimmu.2020.01157 (PMC7326105; doi:10.3389/fimmu.2020.01157)
Supplement: Supplementary file 1 [file Data_Sheet_1.DOCX]

**Supplemental material**

**The receptor for advanced glycation endproducts (RAGE) contributes to severe inflammatory liver injury in mice**

Toni Weinhage, PhD, Timo Wirth, PhD, Paula Schütz, MSc, Philipp Becker, MD, Aloys Lueken, PhD, Boris V. Skryabin, MD, PhD, Helmut Wittkowski, MD, Dirk Foell, MD

**Supplemental methods**

***Generation of Rage-/-*** ***mice***

*RAGE targeting:* For the *Rage* targeting construct a 1.3 kb left flanking region, containing the promoter, exon 1, and 11 nucleotides of the intron 1 genomic sequences, and a 5.2 kb right flanking region containing 37 bp of the intron 7, exons 8 - 11 and 4260 bp of the intron 11 genomic sequences were subcloned from the *Rage* gene containing RP-23 82L12 BAC clone (BACPAC Resources Center (BPRC) Children’s Hospital Oakland Research Institute, USA) with help of the recombineering-based method [15]. A 1.9 kb neomycin cassette flanked by two LoxP sites, was cloned as *Eco*RI – *Bam*HI DNA fragment in between. The pBluescript based plasmid backbone together with the negative selection marker (thymidine kinase cassette), was added to the left flanking region as *Not*I - *Sal*I DNA fragment.

*ES cell transfection:* CV19 ES cells (129Sv x C57BL/6J) were expanded in HEPES-buffered Dulbecco’s modified Eagle medium (DMEM) containing 15% FCS (PAA), 0.1 mM non-essential amino acids, 2 mM L-glutamine, 0.1 mM β-mercaptoethanol, 1000 U/ml of recombinant LIF (MERCK Millipore), 100 U/ml penicillin and 100 µg/ml streptomycin. 2 x 10^7^ cells in 0,8 ml Capecchi buffer [16] were electroporated at 25 µF and 400V (Gene Pulser; Bio-Rad) with 55 µg of linearized targeting vector (pRAGE_targ) DNA. Subsequently, cells were cultivated for 10 min at room temperature and plated onto ten 100-mm diameter culture dishes containing a gamma-irradiated monolayer of mouse primary G418-resistant fibroblast feeder cells. Selection was achieved by adding 350 µg of G418 (Invitrogen) per ml and 0.2 µM 2’-deoxy-2’-fluoro-β-D-arabinofuranosyl-5-iodouracil (FIAU) (Moravek Biochemicals and Radiochemicals, USA) and medium was replaced daily. Positive colonies were picked and analyzed 8 days after plating using the PCR screening, and Southern-blot DNA method using a ^32^P-labeled 1.0 -kb probe containing sequences 5’ to the targeted homology (**Supplemental Figure 1**).

*Generation of mice:* Positive ES cells were injected into 3.5-day B6D2F1 blastocysts and subsequently transferred into the uteri of 2.5-day pseudopregnant CD-1 foster mice. Male chimaeras were crossed to the C57BL/6J female mice. Heterozygous agouti offsprings were confirmed by Southern blot analysis and tested by PCR for the presence of the targeted allele. Cre-mediated excision of the neo cassette was performed *in vivo* by cross-breeding mice harboring the a PGK-1 promoter driven Cre transgene [17] resulting in total heterozygous *Rage*-deficient mice (*Rage +/-*). Subsequently, heterozygous mice were interbred to *Rage-/-* homozygosity.

**
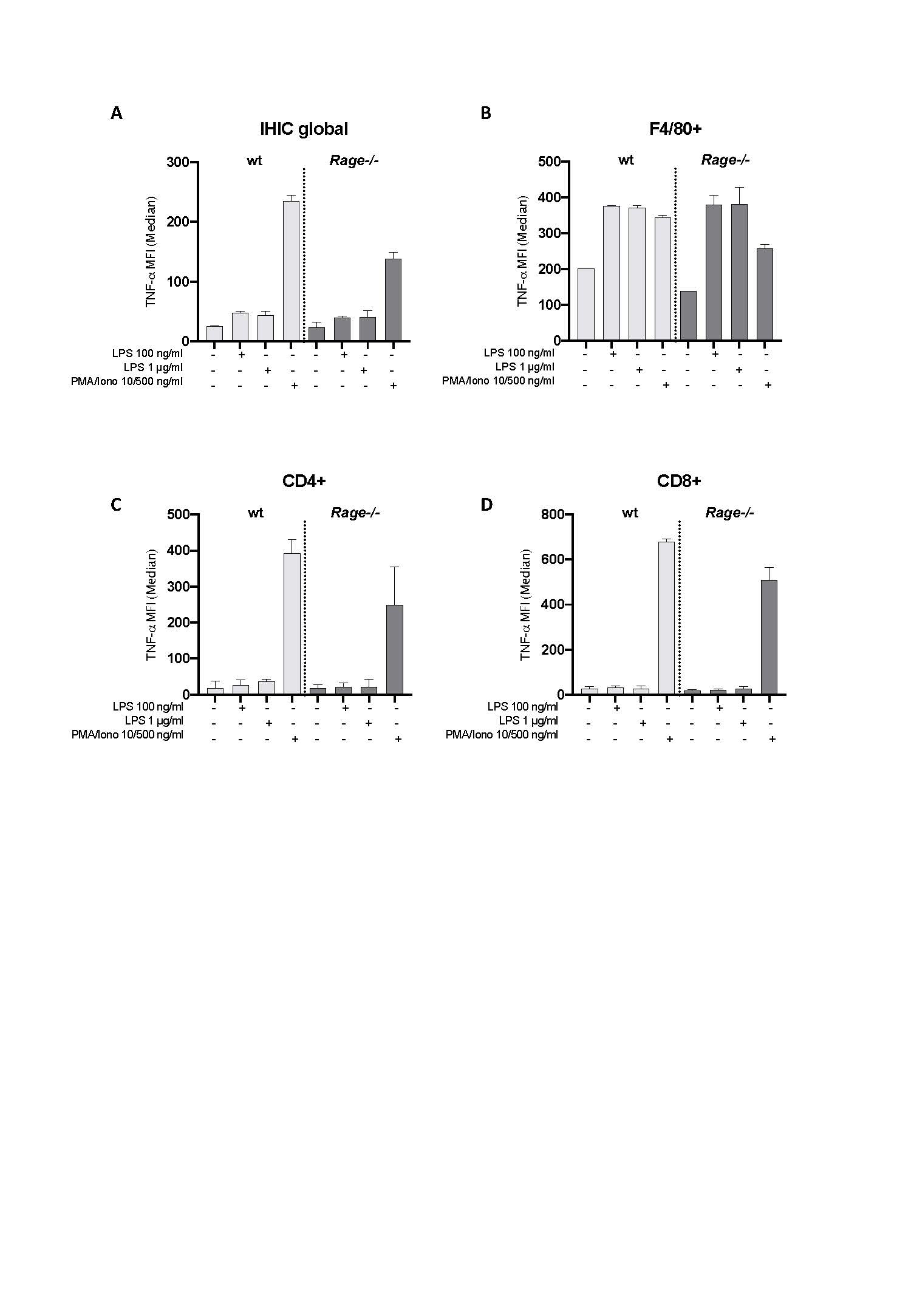
**

**Suppl. Figure 2. Characterization of intrahepatic immune cells (IHICs).**

Primary liver cells were generated by established protocols. IHICs were isolated and subsequently stimulated with LPS or PMA/Ionomycin as a positive control for 4 hours in the presence of a protein transport inhibitor, followed by intracellular staining and FACS analyses to quantify intracellular TNF-α production. **(A)** Quantification of intracellular TNF-α production in IHIC isolated from *Rage-/-* and wildtype (wt) mice. **(B)** Quantification of intracellular TNF-α production in F4/80+/CD11b+ cells (resident macrophages/Kupffer cells). **(C,D)** Quantification of intracellular TNF-α production in CD4+ or CD8+ T lymphocytes. Data are depicted as mean ±SEM of two experiments (n=4 per group). Data are expressed as the median ± SEM of two experiments (n=4 per group). LPS=lipopolysaccharide, D-Gal=D-galN, D-galactosamine.

**Suppl. Table 1:** Antibodies used for flow cytometry

| **Target** | **Clone** | **Manufacturer** |
| --- | --- | --- |
| CD45 | 30-F11 | Biolegend (San Diego, CA, USA) |
| CD11b | M1/70 | Biolegend |
| Ly6G | 1A8 | Biolegend |
| TNF-α | MP6-XT22 | eBioscience (San Diego, CA, USA) |
| Histone H3 (citrulline R2,R8,R17) | polyclonal | Abcam |
| MPO R-PE | 8F4 | Hycult Biotech |
| CD3 | 145-2C11 | Biolegend |
| CD4 | GK1.5 | Biolegend |
| CD8 | 53-6.7 | Biolegend |
| Ly6C | HK1.4 | Biolegend |
| F4/80 | BM8 | Biolegend |
|  |  |  |

**Suppl. Table 2:** Primer sequences used for qRT-PCR

| **Target** | **Forward Primer Sequence (5´-3´)** | **Reverse Primer Sequence (5´-3´)** |
| --- | --- | --- |
| RPS9 | CTGGACGAGGGCAAGATGAAGC | TGACGTTGGCGGATGAGCACA |
| β-actin | GTGACGTTGACATCCGTAAAGA | GCCGGACTCATCGTACTCC |
| HPRT | TCAGTCAACGGGGGACATAAA | GGGGCTGTACTGCTTAACCAG |
| GAPDH | TGACCTCAACTACATGGTCTACA | CTTCCCATTCTCGGCCTTG |
| TNF-α | CACAGAAAGCATGATCCGCGAC | TGCCACAAGCAGGAATGAGAAGAG |
| IL-6 | TAGTCCTTCCTACCCCAATTTCC | TTGGTCCTTAGCCACTCCTTC |
| IL1-β | TGTCTTGGCCGAGGACTAAGG | TGGGCTGGACTGTTTCTAATGC |
| HMGB1 | AGGATCTCCTTTGCCCATGT | TGAGCTCCATAGAGACAGCG |
| RAGE | CCAATGGTTCCCTCCTCCTT | TAAGTGCCAGCTAAGGGTCC |

**Suppl. Table 3:** Histopathological damage score

|  | **Score** |  |  |  |
| --- | --- | --- | --- | --- |
| **Pathology** | **0** | **1** | **2** | **3** |
| hepatocellular necrosis | none | <25% of all the hepatocytes | >25 to 50% | >50% |
| small vacuolisation and / or cell lysis | none | <25% | >25% |  |
| accumulation of erythrocytes in the sinusoids | none | minimal | moderate | severe |
| Neutrophil infiltration | none | <3 vessel | >3 vessel | extravasal |
